# The relative magnitude of transgene-specific adaptive immune responses induced by human and chimpanzee adenovirus vectors differs between laboratory animals and a target species

**DOI:** 10.1016/j.vaccine.2015.01.042

**Published:** 2015-02-25

**Authors:** Matthew D.J. Dicks, Efrain Guzman, Alexandra J. Spencer, Sarah C. Gilbert, Bryan Charleston, Adrian V.S. Hill, Matthew G. Cottingham

**Affiliations:** a The Jenner Institute, University of Oxford, ORCRB, Roosevelt Drive, Oxford OX3 7DQ, UK; b The Pirbright Institute, Pirbright Laboratory, Pirbright, Surrey GU24 0NF, UK

**Keywords:** Viral vector, Vaccine immunology

## Abstract

Adenovirus vaccine vectors generated from new viral serotypes are routinely screened in pre-clinical laboratory animal models to identify the most immunogenic and efficacious candidates for further evaluation in clinical human and veterinary settings. Here, we show that studies in a laboratory species do not necessarily predict the hierarchy of vector performance in other mammals. In mice, after intramuscular immunization, HAdV-5 (*Human adenovirus C*) based vectors elicited cellular and humoral adaptive responses of higher magnitudes compared to the chimpanzee adenovirus vectors ChAdOx1 and AdC68 from species *Human adenovirus E*. After HAdV-5 vaccination, transgene specific IFN-γ^+^ CD8^+^ T cell responses reached peak magnitude later than after ChAdOx1 and AdC68 vaccination, and exhibited a slower contraction to a memory phenotype. In cattle, cellular and humoral immune responses were at least equivalent, if not higher, in magnitude after ChAdOx1 vaccination compared to HAdV-5. Though we have not tested protective efficacy in a disease model, these findings have important implications for the selection of candidate vectors for further evaluation. We propose that vaccines based on ChAdOx1 or other *Human adenovirus E* serotypes could be at least as immunogenic as current licensed bovine vaccines based on HAdV-5.

## Highlights

• HAdV-5 (HAdV-C) vectors are more immunogenic than AdC68 or ChAdOx1 (HAdV-E) vectors in mice.
• In mice, CD8^+^ T cell responses peak later, and are more durable after HAdV-5 vaccination.
• In cattle, ChAdOx1 is at least as immunogenic as HAdV-5.

## 1 Introduction

Replication-defective recombinant adenoviruses are now widely employed as vectors in the development of both human and veterinary vaccines [14,16,40]. Pre-existing neutralising antibodies to common human adenoviruses such as human adenovirus serotype 5 (HAdV-5) may have contributed to the lack of clinical efficacy of some vaccine vectors based on this serotype in human trials [21]. Adenoviruses isolated from chimpanzees and other great apes group phylogenetically within the human adenovirus species [32] but the seroprevalence of neutralising antibodies against these serotypes in humans is considerably lower than against HAdV-5, prompting the development of chimpanzee adenoviruses (ChAds) as vaccine vectors [8,42]. ChAd vectors have primed unprecedented frequencies of antigen-specific CD8^+^ T cells in recent human clinical vaccine trials [26,35,36].

For veterinary applications, HAdV-5 is still the most commonly used human adenovirus serotype. Indeed, a new foot-and-mouth disease virus (FMDV) vaccine based on this serotype has recently been licensed in the United States for use in cattle [27]. Some studies have successfully used serotypes originating from the target species, such as bovine adenovirus vectors in cattle, despite pre-existing immunity to the vector [46]. However, the use of alternative adenovirus serotypes has yet to be fully explored.

Vector serotypes for both human and veterinary applications are currently screened and selected largely on the basis of pre-clinical studies in mice [8]. However, it remains unclear to what extent these pre-clinical studies predict immunogenicity and efficacy in other mammalian species. In this study we compared antigen-specific immune responses elicited by vaccination with a HAdV-5 vector (*Human adenovirus C*, here referred to as species C) or a new chimpanzee adenovirus vector ChAdOx1 [13] (*Human adenovirus E*, here referred to as species E), in mice and cattle. In cattle, we tested vectors encoding mycobacterial antigen 85A to represent bovine tuberculosis vaccine candidates in the target species [12,43]. This is the first time, to our knowledge, that a chimpanzee adenovirus vector, or any vector derived from species E, has been tested in a ruminant species. In both species, we demonstrate differences in the ability of vectors of different serotypes to elicit cellular and humoral immune responses. Furthermore, by comparing studies in laboratory animals and cattle, we demonstrate that comparative immunogenicity studies in mice do not predict the hierarchy of vector performance in a target species.

## 2 Materials and methods

### 2.1 Viral vectors

Propagation and titration of E1-E3-deleted recombinant HAdV-5, and chimpanzee adenovirus vectors AdC68 and ChAdOx1 on HEK293A cells (Invitrogen) has been described previously [13]. The design of vaccine antigen constructs has been described previously for TIPeGFP (an epitope string, TIP, fused to the N-terminus of enhanced GFP) [1,9], and Influenza A virus NP + M1 (fusion of nucleoprotein and matrix 1 protein sequences joined by a glycine-proline linker) [25]. The *Mycobacterium tuberculosis* (TB) antigen 85A (Ag85A) construct used in this study was a codon-optimized (for mammalian expression) version of the Ag85A construct from TB vaccine candidate MVA-85A [22]. All recombinant vectors expressing TIPeGFP were titered by enumeration of single GFP-positive HEK293 cells by epifluorescent microscopy, while vectors expressing Ag85A and NP + M1 transgenes were titered by immunostaining for the vaccine antigen as described previously [13]. For Ag85A, the monoclonal antibody SV5-PK1 (AbCam) was used to detect a V5 tag fused to the C-terminus. For NP + M1, monoclonal antibody GA2B against Influenza A matrix 1 protein (AbCam) was used. Viral particle estimates were performed by spectrophotometric measurement of absorbance at 260 nm [20]. The ratios of estimated viral particles to infectious particles (P:I ratios) were as follows: HAdV-5 TIPeGFP, 15; ChAdOx1 TIPeGFP, 17; AdC68 TIPeGFP, 32; HAdV-5 Ag85A, 7; ChAdOx1 Ag85A, 27; HAdV-5 NP + M1, 12; ChAdOx1 NP + M1, 38.

### 2.2 Mice and immunizations

Female BALB/c or C57BL/6J mice (Harlan, UK) above 6 weeks of age were immunized intramuscularly (i.m.). Viral vectors were formulated in phosphate buffered saline (PBS, Sigma, UK) in a total volume of 50 μl and injected into the tibialis anterior muscle of each animal. All mouse procedures were performed in accordance with the terms of the UK Animals (Scientific Procedures) Act Project Licence (PPL 30/2414 or PPL 30/2889) and were approved by the University of Oxford Animal Care and Ethical Review Committee.

### 2.3 Mouse immunology

Spleen *ex vivo* interferon-gamma (IFN-γ) ELISpot was performed as described previously [19]. To measure vaccine antigen specific responses, cells were re-stimulated for 18–20 h with peptides (Supplementary Table 1) at a final concentration of 1 μg/mL. Spot forming cells (SFC) were measured using an automated ELISpot reader system (AID). Flow cytometry on peripheral blood mononuclear cells (PBMC) was performed as described previously [37], except that peptide re-stimulation was with 1 μg/mL *Pb*9. All fluorophore-conjugated antibodies were obtained from eBiosciences. Data were acquired on a CyAn-ADP flow cytometer (Dako) and analysed using FlowJo (Treestar). The program SPICE was used to generate graphical representations of functional T cell responses using background-deducted data [31]. IgG endpoint ELISA was performed as described previously [6,15] except plates were coated with recombinant GFP protein (Millipore, UK) at 1 μg/mL.

### 2.4 Cattle immunization

MHC-defined, weight- and age-matched, conventionally reared Friesian Holstein cattle from The Pirbright Institute's (Pirbright) MHC-defined herd were vaccinated i.m. with viral vectors formulated in PBS.

### 2.5 Cattle immunology

The following fluorochrome-labeled mouse anti-bovine monoclonal antibodies (MAb) were used for flow cytometry and have been described previously [10]: CC30-APC/Cy5.5 (anti-CD4), CC63-APC/Cy7 (anti-CD8), ILA-111–Alexa Fluor 610/PE (anti-CD25), CC302-PE (anti-IFN-γ), CC328-PE/Cy5.5 (anti-TNF-α) and Ab86-APC (anti-IL-2, Serotec). All antibodies were obtained from Pirbright except where noted. Dead cells were gated out using LIVE/DEAD aqua or propidium iodide (Invitrogen). Cells were analyzed using an LSR Fortessa (Becton Dickinson), and staining assessed using FCS Express v4 (DeNovo Software). To detect Ag85A-specific antibodies, 96-well plates (Costar) were coated with 0.25 μg of recombinant Ag85A (Lionex, Germany) for 2 h at room temperature. Plates were washed with PBS/Tween and blocked with Sea Block (Pierce) overnight, washed again with PBS/Tween and dilutions of sera added to the plate. After 1 h, the plates were washed and rabbit anti-bovine HRP (Serotec) was added. The plates were incubated for 1 h, washed and TMB substrate (Pierce) added. After a 15 min incubation the reactions were stopped with 1 M H_2_SO_4_ and plates read on a Fluorostar Optima (BMG Labtech, Germany).

### 2.6 Statistical analyses

Statistical analyses as indicated in figure legends were performed using Prism (GraphPad Software, Inc.).

## 3 Results

### 3.1 HAdV-5 vectors elicit higher frequencies of vaccine antigen specific T cells and antibodies in mice than AdC68 and ChAdOx1 based vectors

The ability of AdC68 and ChAdOx1 to induce transgene product specific T cell and antibody responses was compared with HAdV-5 in a series of dose-response experiments in mice with vectors encoding the model antigen TIPeGFP [1]. Responses were assessed after two weeks, identified previously to be at or near the peak of the antigen specific CD8^+^ T cell response after adenovirus vector administration [4,38]. HAdV-5 elicited higher frequencies of splenic transgene product specific IFN-γ^+^ CD8^+^ and CD4^+^ T cell responses than either of the chimpanzee adenovirus vectors in the 10^5^–10^8^ infectious units (ifu) dose range (Fig. 1A–C). The two chimpanzee adenovirus vectors induced similar CD8^+^ and CD4^+^ T cell frequencies, consistent with our previous studies [13]. GFP-specific total IgG antibody titers at the maximum 10^8^ ifu dose were approximately two orders of magnitude higher after HAdV-5 than after ChAdOx1 or AdC68 (Fig. 1D). Titers were below the limit of detection after administration of either ChAd vector at doses lower than 10^8^ ifu, though detectable titers were still observed after vaccination with 10^6^ ifu HAdV-5 (data not shown). Higher transgene specific CD8^+^ T cell frequencies (at 10^6^ ifu) and IgG endpoint titers (at 10^8^ ifu) were also observed in C57BL/6 mice after vaccination with HAdV-5 TIPeGFP than with ChAdOx1 TIPeGFP (Supplementary Fig. 1).

We compared the CD8^+^ T cell responses elicited by HAdV-5 and ChAdOx1 vectors encoding antigen constructs that have been employed in clinical trials: 85A [12,39,43] and NP + M1 [2]. IFN-γ ELISpot responses to the epitope P11 were of higher frequency after HAdV-5 85A administration than after ChAdOx1 85A at a dose of 10^8^ ifu (*p* < 0.001) (Fig. 2A). Recombinant HAdV-5 NP + M1 elicited a higher frequency of CD8^+^ T cells against the immunodominant epitope NP_147–155_ than ChAdOx1 NP + M1 at a dose of 10^6^ (*p* < 0.001) but not at doses of 10^7^ or 10^8^ ifu (Fig. 2B).

### 3.2 The IFN-γ^+^ CD8^+^ T cell response after HAdV-5 vaccination exhibits a later peak magnitude and contraction to memory than after AdC68 and ChAdOx1 vaccination

IFN-γ^+^ CD8^+^ T cell responses were monitored longitudinally by intracellular cytokine staining (ICS) of peripheral blood mononuclear cells (PBMCs). Fig. 3A shows that, after an intramuscular dose of 10^8^ ifu, HAdV-5 TIPeGFP elicited a higher frequency of *Pb*9 specific CD8^+^ T cell responses throughout the eight-week duration of study than AdC68 and ChAdOx1 vectors expressing the same recombinant antigen. The peak magnitude of the response after HAdV-5 administration was at the three-week time-point, while the peak of the response after vaccination with either ChAd vector was at, or prior to, the two-week time-point.

To assess memory phenotype, PBMCs were stained for the surface markers CD62L and CD127 to define effector T cells (T_EFF_), effector memory T cells (T_EM_), and central memory T cells (T_CM_) as described previously [3] (Fig. 3B). Although the percentage of *Pb*9 specific CD8^+^ T cells displaying a T_EFF_ phenotype decreased with time in all three groups (consistent with a gradual differentiation to T_EM_), HAdV-5 vaccination elicited an increased T_EFF_ response compared with AdC68 and ChAdOx1 up to 35 days post-vaccination. However, 56 days post-vaccination, the relative proportions of T_EFF_ and T_EM_ cells were comparable between all three groups, despite the overall frequency of *Pb*9 specific IFN-γ^+^ CD8^+^ T cells being statistically significantly higher in the HAdV-5-vaccinated group (Fig. 3B).

At a 100-fold lower dose (10^6^ ifu), there was a trend towards higher responses after HAdV-5 vaccination, but IFN-γ^+^ CD8^+^ T cell frequencies were not significantly different from those in the AdC68-vaccinated group (Fig. 3C). Memory phenotypes could not be determined reliably owing to the limited size of the *Pb*9-specific CD8^+^ T cell population.

### 3.3 Cytokine secretion by vaccine antigen-specific CD8^+^ T cells varies with time after vaccination with HAdV-5 versus ChAdOx1

Production of IFN-γ, TNF-α and IL-2 by *Pb*9 specific CD8^+^ T cells was assessed by ICS of PBMCs collected from the same animals used in the experiment shown in Fig. 3. Twelve days post vaccination, a significantly higher percentage of cells produced more than one of these three cytokines after HAdV-5 vaccination than after AdC68 or ChAdOx1 (Fig. 4). The percentage of cells expressing all three cytokines was also significantly higher in the HAdV-5 vaccinated group. At 21 days and 35 days post-vaccination, the percentage of cells expressing more than one cytokine remained significantly higher in the HAdV-5 vaccinated group. At 56 days post-vaccination, cytokine production profiles were comparable in groups vaccinated with HAdV-5 and ChAdOx1, though an elevated percentage of cells expressing only one of the three cytokines was maintained in the AdC68 vaccinated group (Fig. 4).

### 3.4 ChAdOx1 elicits higher vaccine antigen specific T cell frequencies and antibody titers than HAdV-5 after intramuscular administration in cattle

Transgene product-specific T cell and antibody responses were compared after intramuscular injection of cattle with 10^9^ ifu of HAdV-5 85A or ChAdOx1 85A vectors. Fig. 5A shows that 85A-specific IFN-γ^+^ CD8^+^ T cell frequencies in the blood were highest at the two-week time-point and were statistically significantly higher at this time-point after ChAdOx1 than after HAdV-5 vaccination. 85A-specific IFN-γ^+^ CD4^+^ T cell responses followed a similar kinetic, albeit at lower frequencies than the CD8^+^ T cell responses, and the difference in frequency between vectors was not statistically significant at any time-point (Fig. 5B). 85A-specific serum IgG antibody titers continued to rise during the first four weeks after vaccination with both vectors (Fig. 5C). There was a trend toward higher anti-85A endpoint titers after ChAdOx1 vaccination throughout the six-week duration of study, but statistical significance was reached at the two-week time-point only.

### 3.5 Cytokine production profiles of vaccine antigen specific CD8^+^ T cells are comparable after vaccination with HAdV-5 and ChAdOx1 vectors in cattle

Production of IFN-γ, TNF-α and IL-2 by 85A-specific CD8^+^ T cells at the two-week and three-week time-points was assessed by ICS of PBMCs collected from the same animals used in the experiment shown in Fig. 5. The data shown in Fig. 6 indicate that the profiles of cytokine secretion of induced CD8^+^ T cells were similar between ChAdOx1 and HAdV-5 vaccinated animals at both these time points. There were no statistically significant differences in the percentages of 85A -specific CD8^+^ T cells producing one, two or three cytokines between the two and three week time points.

## 4 Discussion

This study revealed marked differences in the relative magnitude of transgene product specific T cell and antibody responses elicited by human and chimpanzee adenovirus vectors in two mammalian species. In mice, HAdV-5 elicited T cell and antibody responses were higher in frequency than HAdV-E chimpanzee adenovirus vectors after intramuscular immunization. This relationship was observed using three antigens and in two mouse strains, suggesting that it is independent of the transgene or inbred mouse strain. In cattle, an important target of veterinary vaccination, a different relationship was observed; ChAdOx1 was as immunogenic as HAdV-5.

In mice, the observation that HAdV-5 vectors are more immunogenic than HAdV-E ChAd vectors is consistent with some published studies [7,8,29] but is not supported by others [28,30,38]. Disagreements within the published literature may result from differences in the method used to determine vaccine dose [13]. Previous publications that are not consistent with the current study based vaccine doses on viral particle estimation rather than on infectious titers.

The relationship between vector dose and the magnitude of CD8^+^ T cell responses varied considerably with different transgene antigens or epitopes, highlighting the importance of conducting comparative studies with multiple antigens across a range of doses (Figs. 1 and 2). Mechanisms responsible for differences in the magnitude of responses against different transgene antigens are not well understood, but likely involve differences in the efficiency of antigen expression, peptide processing and presentation on MHC molecules, and the hierarchy of immunodominant epitopes present within both the viral vector and antigen construct [33,45]. Previous studies comparing vectors in mice have typically administered vaccine doses in the order of 10^10^ viral particles (equivalent to 5 × 10^8^–5 × 10^9^ ifu): at high doses, potential differences in immunogenicity between vectors may be undetectable as the magnitudes of responses reach a plateau. At a dose of 10^8^ ifu, *Pb*9 specific IFN-γ^+^ CD8^+^ T cell responses reached peak magnitude later and were more durable after HAdV-5 vaccination compared to ChAdOx1 and AdC68. However, at a 100-fold lower dose, the magnitude and kinetic of the *Pb*9 specific CD8^+^ T cell response was comparable between all three vectors (Fig. 3C) demonstrating a dependence on vector dose, as previously suggested [29]. We also observed a later contraction to memory after HAdV-5, with a significantly higher proportion of CD8^+^ T cells retaining an effector (T_EFF_) phenotype compared to the ChAd vectors. After eight weeks, the proportion of T_EFF_ relative to effector memory CD8^+^ T cells (T_EM_) was similar for all three vectors, with a majority adopting a T_EM_ phenotype as observed previously [30,44].

Few previous studies have assessed the kinetics of vaccine antigen specific T cell responses after adenovirus vector administration in cattle [43], and none to our knowledge have compared the immunogenicity of vectors derived from members of different human adenovirus species. In the current study, IFN-γ^+^ CD8^+^ and CD4^+^ T cell responses were highest two weeks post-vaccination (Fig. 5), similar to observations in mice at the lower 10^6^ ifu dose (Fig. 3C). We have not investigated the effect of dose on the timing of peak CD8^+^ T cell frequency in cattle. Both HAdV-5 and ChAdOx1 induced higher CD8^+^ T cell responses than CD4^+^ T cell responses, in agreement with data in mice from this study and the consensus in the literature [18,40]. Antibody titers continued to increase at least six weeks after immunization of both vectors. The continued elevation could suggest that expression of the transgene product is persistent, as in mice [41], although studies in cattle with different antigens have observed antibody titers reaching a plateau after just two weeks [24] and a marked reduction in transgene expression at the injection site after 24 h [23]. The kinetics of CD8^+^ T cell, CD4^+^ T cell, and antibody responses were similar after HAdV-5 and ChAdOx1 vaccination, perhaps a function of the equivalence in magnitude of these responses in cattle.

Recent studies have suggested that the ability of primed CD8^+^ and CD4^+^ T cells to secrete multiple cytokines may be an important requirement for establishing a protective immune response [5,11,34]. In mice, a higher proportion of CD8^+^ T cells primed after HAdV-5 vaccination secreted two or more of the three cytokines IFN-γ, TNF-α and IL-2 during the first 35 days post vaccination compared to AdC68 and ChAdOx1 vaccinated animals (Fig. 4). In cattle, however, there were no statistically significant differences in cytokine secretion profiles between CD8^+^ T cells primed after HAdV-5 or ChAdOx1 vaccination (Fig. 6).

It is unclear whether the differences in frequency and phenotype of vaccine antigen specific T cells observed in this study are due to differences in intrinsic vector biology including activation of distinct innate signalling pathways, or simply due to differences in the effective antigen dose delivered by HAdV-5 and ChAd vectors. In mice, a previous study has shown that HAdV-5 elicits higher transgene expression *in vivo* after vaccination and within murine DCs transduced *in vitro* compared to AdC68 [17]. In this study, the kinetic of the CD8^+^ T cell response in mice was dose dependent, and HAdV-5 responses at 10^6^ ifu followed a similar kinetic to ChAd induced responses at 10^8^ ifu. Persistence of antigen after HAdV-5 administration at the 10^8^ ifu dose may be responsible for the observed differences in kinetic and for the delayed contraction of CD8^+^ T cells to a T_EM_ phenotype. Indeed, the proportion of transgene specific CD8^+^ T cells expressing CD127 has been shown to decrease with increasing dose of HAdV-5 [29]. Differences in the proportion of T cells secreting multiple cytokines may also be a function of the overall magnitude of the response. In cattle, CD8^+^ T cell responses were similar in magnitude between HAdV-5 and ChAdOx1, and no significant differences in T cell ‘multi-functionality’ were observed.

At present, selection of adenovirus vectors for human and veterinary applications is largely based on comparative studies in mice [8]. Here, we show that the hierarchy of vector immunogenicity in mice does not predict relative immunogenicity in cattle. Vectors derived from members of species E, including those isolated from chimpanzees, represent promising alternatives to current HAdV-5 based bovine vaccine platforms.

## Conflict of interest statement

MDJD, SCG, AVSH, and MGC are named inventors on a patent application describing the ChAdOx1 vector (PCT Application No. PCT/GB2012/000467).

## Figures and Tables

**Figure:**
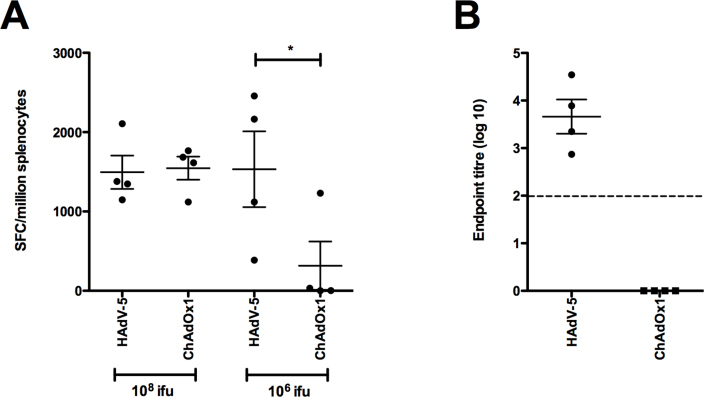


**Fig. 1:**
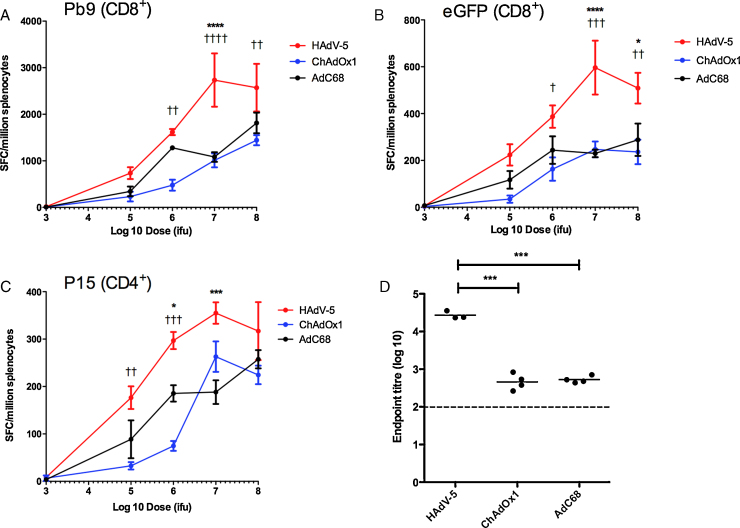
*TIPeGFP specific IFN-γ^+^ T cell frequencies and antibody titers are higher after vaccination of mice with HAdV-5 than with AdC68 or ChAdOx1.* BALB/c mice (4/group) were immunised i.m. with 10^3^–10^8^ ifu of HAdV-5 TIPeGFP, AdC68 TIPeGFP, or ChAdOx1 TIPeGFP. Two weeks post vaccination, splenic T cell responses against peptide epitopes (A) Pb9 (CD8^+^), (B) EGFP_200–208_ (CD8^+^) and (C) P15 (CD4^+^) were measured by IFN-γ ELISpot. Note that Pb9 and P15 epitopes are encoded within the TIP epitope string of TIPeGFP. Graphs show mean response and SEM. Statistical analysis performed by two-way ANOVA with Bonferroni multiple comparison post-test. HAdV-5 versus AdC68; **** *p* < 0.0001, *** *p* < 0.01, * *p* < 0.05. HAdV-5 verses ChAdOx1; †††† *p* < 0.0001, ††† *p* < 0.001, †† *p* < 0.01, † *p* < 0.05. Overall ANOVA *p*-value for effect of vector <0.0001 in both A and B and <0.001 in C. Experiments with each vector were performed separately but included a group vaccinated with 10^6^ ifu HAdV-5 TIPeGFP (approximately the ED_50_) to assess variability across experiments. No statistically significant differences in responses were observed in this control group across individual experiments. (D) Anti-GFP IgG titers in the serum of the same mice measured by endpoint ELISA. Dashed lines in D indicate limit of detection of the assay. Statistical analysis performed by one-way ANOVA with Bonferroni post-test.

**Fig. 2:**
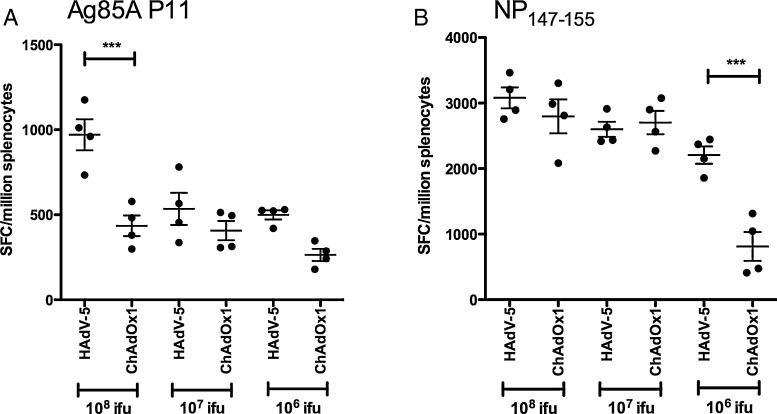
*HAdV-5 vaccination elicits higher transgene-product specific CD8^+^ T cell frequencies than ChAdOx1 with Ag85A and NP* *+* *M1 antigen constructs*. (A) BALB/c mice were immunised intramuscularly with 10^6^–10^8^ ifu HAdV-5 85A or ChAdOx1 85A. Splenic T cell responses against epitope P11 were measured by IFN-γ ELISpot 2 weeks post-vaccination. (B) BALB/c mice were immunised intramuscularly with 10^6^–10^8^ ifu HAdV-5 NP + M1 or ChAdOx1 NP + M1. Splenic IFN-γ ELISpot responses against epitope NP_147-155_ were measured two weeks post vaccination. Mean response indicated, with error bars showing SEM. Statistical analysis performed by two-way ANOVA with Bonferroni post-test. *** *p* < 0.001. Overall ANOVA *p*-value for effect of vector in A; *p* < 0.0001 B; *p* = 0.0026.

**Fig. 3:**
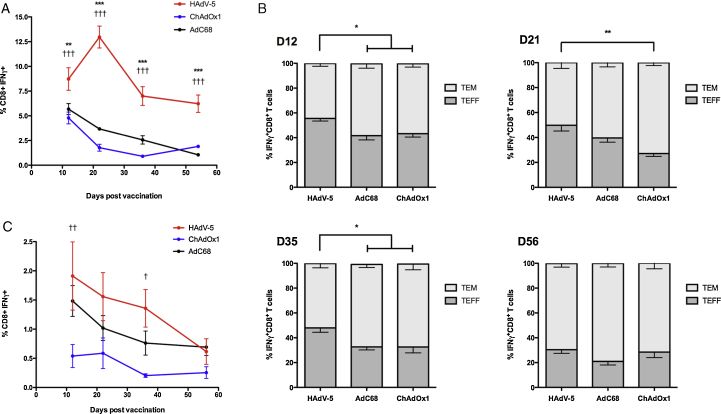
*IFN-γ^+^CD8^+^ T cell responses in mice after HAdV-5 vaccination peak later and are more durable than after AdC68 or ChAdOx1 vaccination.* (A)–(B) BALB/c mice (6/group) were immunised with 10^8^ ifu of HAdV-5 TIPeGFP, AdC68 TIPeGFP or ChAdOx1 TIPeGFP. Blood was collected on days 12, 21, 36, and 56 post vaccination and *Pb*9 specific CD8^+^ IFN-γ^+^ T cell responses were measured by ICS. (A) *Pb*9 specific IFN-γ^+^CD8^+^ T cells as a percentage of total CD8^+^ T cells. Graphs display mean and SEM. Statistical analysis by two-way repeated measures ANOVA with Bonferroni post tests. HAdV-5 versus AdC68; *** *p* < 0.001, ** *p* < 0.01. HAdV-5 vs ChAdOx1; ††† *p* < 0.001 (B) *Pb*9 specific CD8^+^ IFN-γ^+^ T cells from the same mice as in A were identified as T_EFF_ (CD127^−^CD62L^−^) or T_EM_ (CD127^+^CD62L^−^), and the frequency of each subset was calculated as a percentage of the total response. Frequencies of CD8^+^ T_CM_ cells (CD127^+^CD62L^+^) were <1% of the total IFN-γ^+^CD8^+^ T cell response at all time points. Stacked bars display mean and SEM; statistical analysis by one-way ANOVA at each time point. ** *p* < 0.01, * *p* < 0.05. (C) The experiment in A-B was repeated using doses of 10^6^ ifu. HAdV-5 vs ChAdOx1; †† *p* < 0.01, † *p* < 0.05. No statistically significant differences between HAdV-5 and AdC68 groups.

**Fig. 4:**
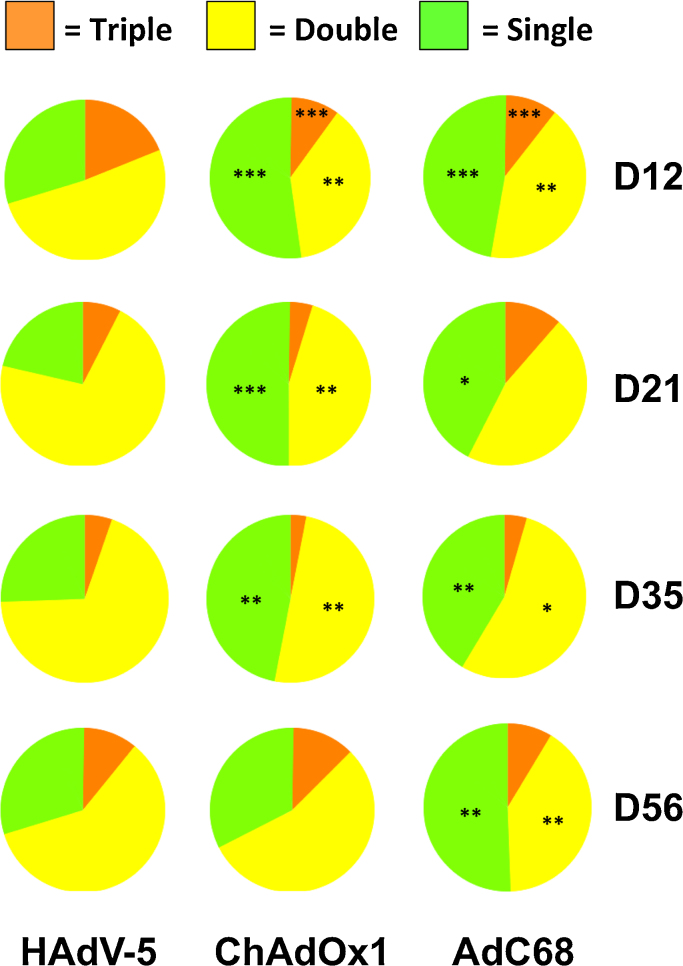
*Profile of cytokine expression by CD8^+^ T cells in mice after vaccination with HAdV-5, ChAdOx1 and AdC68 vectors varies over time.* ICS of *Pb*9-specific CD8^+^ T cells (from the same experiment as Fig. 3A and B) for IFN-γ, IL-2, and TNF-α. The proportion of CD8^+^ T cells producing all three cytokines (orange), any combination of two cytokines (yellow), or any one cytokine only (green), in response to peptide re-stimulation is show in the pie charts below. Statistical analyses on pie chart segments were performed by one-way ANOVA with Bonferroni post-tests. Segments from ChAdOx1 and AdC68 groups that differ statistically significantly in percentage from the corresponding segment in the HAdV-5 group are indicated. *** *p* < 0.001,** *p* < 0.01, * *p* < 0.05. (For interpretation of the references to color in this figure legend, the reader is referred to the web version of this article.)

**Fig. 5:**
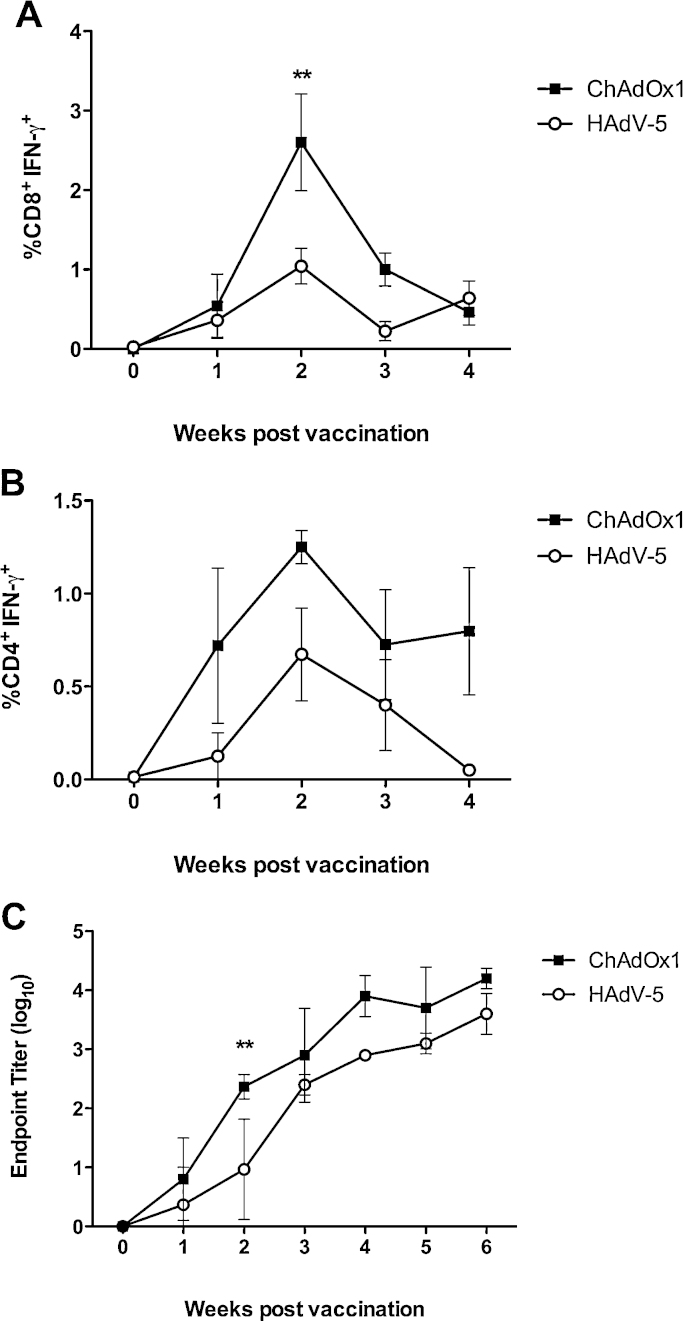
*ChAdOx1 elicits higher transgene product specific T cell frequencies and IgG antibody titers than HAdV-5 after intramuscular vaccination of cattle.* Friesian Holstein cattle (4/group) were immunized intramuscularly with 10^9^ ifu of HAdV-5-85A or ChAdOx1-85A. Blood samples were taken prior to immunization (week 0), and then once per week for a further six weeks. 85A-specific CD8^+^ T cell (A) and CD4^+^ T cell (B) responses were measured by ICS. (C) Anti-85A IgG titers were assessed by endpoint ELISA. Statistical analyses were performed by two-way repeated measures ANOVA between the two groups at each time point (**p < 0.01).

**Fig. 6:**
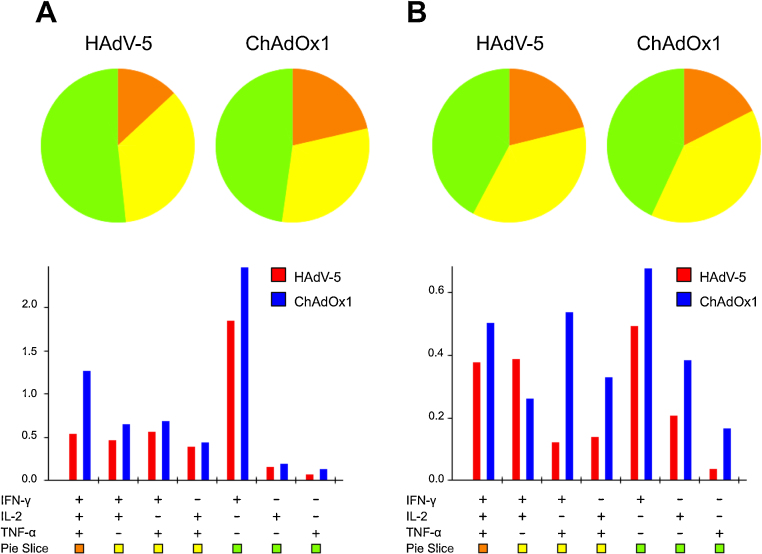
*Similar profiles of expression of three cytokines by CD8^+^ T cells in cattle vaccinated with HAdV-5 and ChAdOx1.* ICS of 85A-specific CD8^+^ T cells (from the same experiment as Fig. 5) for IFN-γ, IL-2, and TNF-α at (A) two weeks or (B) three weeks post-vaccination. The proportion of CD8^+^ T cells producing all three cytokines (orange), any combination of two cytokines (yellow), or any one cytokine only (green), in response to peptide re-stimulation is shown in the pie charts. Bar charts indicate the percentages of total CD8^+^ T cells secreting each combination of cytokines. Statistical analyses were performed by two-tailed *t*-tests between groups at each time-point; there were no significant differences at either time-point. There were also no statistically significant differences in the percentages of 85A-specific CD8^+^ T cells producing one, two or three cytokines between the two and three week time points by one-way ANOVA. (For interpretation of the references to color in this figure legend, the reader is referred to the web version of this article.)
