# Supplementary material for: The relative magnitude of transgene-specific adaptive immune responses induced by human and chimpanzee adenovirus vectors differs between laboratory animals and a target species
Source: Vaccine. 2015 Feb 25;33(9):1121–8. doi: 10.1016/j.vaccine.2015.01.042 (PMC4331283; doi:10.1016/j.vaccine.2015.01.042)
Supplement: Supplementary file 1 [file mmc2.docx]

**Supplementary Table 1. Peptide epitopes used in this study**

| **Peptide** | **MHC restriction** | **Vaccine antigen** | **Origin** | **Sequence** | **Reference** |
| --- | --- | --- | --- | --- | --- |
| *Pb*9 | CD8^+^ (H2-K^d^) | TIPeGFP | *Plasmodium berghei* CSP | SYIPSAEKI | ([34](#_ENREF_34)) |
| EGFP_200-208_ | CD8^+^ (H2-K^d^) | TIPeGFP | GFP | HYLSTQSAL | ([18](#_ENREF_18)) |
| P15 | CD4^+^ (H-2-d) | TIPeGFP / Ag85A | *Mycobacterium tuberculosis* 85A | MTFLTSELPGWLQANRHVKPT | ([24](#_ENREF_24)) |
| AL11 | CD8^+^ (H2-D^b^) | TIPeGFP | SIV-Gag | AAVKNWMTQTL | ([4](#_ENREF_4)) |
| P11 | CD8^+^ (H2-K^d^) | Ag85A | *Mycobacterium tuberculosis* 85A | WYDQSGLSV | ([24](#_ENREF_24)) |
| NP_147-155_ | CD8^+^ (H2-K^d^) | Influenza A NP+M1 | Influenza A | TYQRTRALV | ([35](#_ENREF_35)) |
